# Disentangling Procedural and Patient-Specific Drivers of Perioperative Outcomes in Pelvic Organ Prolapse Surgery: A Stratified Multigroup Analysis

**DOI:** 10.3390/healthcare14121676

**Published:** 2026-06-12

**Authors:** Diana Pop-Lodromanean, Nicolae Grigore, Adrian Hasegan, Samuel Bogdan Todor, Paula Anderco, Radu Chicea, Cristian Ichim, Livia-Mirela Popa

**Affiliations:** Faculty of Medicine, Lucian Blaga University of Sibiu, 550169 Sibiu, Romania; didi_lodro@yahoo.com (D.P.-L.); nicolae.grigore@ulbsibiu.ro (N.G.); adrian.hasegan@ulbsibiu.ro (A.H.); radu.chicea@ulbsibiu.ro (R.C.); cristian.ichim@ulbsibiu.ro (C.I.); liviamirelapopa@yahoo.com (L.-M.P.)

**Keywords:** pelvic organ prolapse, pelvic floor surgery, perioperative complications, risk factors

## Abstract

**Background:** Perioperative outcomes in pelvic organ prolapse (POP) surgery remain difficult to predict due to substantial heterogeneity in both surgical techniques and patient characteristics. Existing studies typically evaluate these factors in isolation, limiting their ability to support individualized risk stratification. This study introduces a stratified analytical framework to disentangle the relative impact of procedural and patient-related determinants across common vaginal reconstructive approaches. **Methods:** A retrospective cohort of 376 women undergoing POP surgery between 2020 and 2025 was analyzed. Patients were stratified into three procedure groups: sacrospinous fixation with mid-urethral sling (SFM + TOT/TVT), anterior and posterior repair with sling (A&P + TOT/TVT), and isolated anterior and posterior repair (A&P alone). Key outcomes included intraoperative blood loss, length of hospitalization, postoperative hospital stay and catheterization time. Within-group predictors were assessed using stratified odds ratios and synthesized via a random-effects model. **Results:** Procedure type was consistently associated with recovery-related outcomes, although it explained only a modest proportion of outcome variability. Patients undergoing A&P repair exhibited significantly prolonged hospitalization (8.00 vs. 6.29 and 6.94 days), postoperative recovery (4.99 vs. 3.48 and 4.17 days), and catheterization duration (3.31 vs. 2.33 and 2.86 days) (all *p* < 0.001). In contrast, intraoperative blood loss was primarily driven by patient-specific factors, including concomitant hysterectomy, prolapse severity, obesity, age, and obstetric history. Prolonged hospitalization was strongly associated with combined procedural complexity and clinical burden, while catheterization duration was influenced by postoperative complications and parity. **Conclusions:** This study demonstrates that perioperative outcomes in POP surgery arise from distinct and interacting domains: procedural factors predominantly shape recovery trajectories, whereas patient characteristics govern intraoperative risk. The proposed stratified random-effects framework enables integrated evaluation across heterogeneous surgical groups and provides an exploratory basis for identifying domains that may inform future individualized perioperative risk models.

## 1. Introduction

Pelvic organ prolapse (POP) is a highly prevalent pelvic floor disorder affecting women predominantly in later life and representing a major source of gynecological morbidity [1]. Pelvic organ prolapse occurs as a result of compromised pelvic support structures, permitting the downward displacement of pelvic organs into the vaginal cavity. This leads to anatomical alterations accompanied by symptoms such as pelvic discomfort, urinary disturbances, and sexual dysfunction, significantly affecting quality of life and frequently necessitating reconstructive pelvic surgery [2,3].

The pathophysiology of pelvic organ prolapse is multifactorial, encompassing mechanical influences alongside alterations in connective tissue integrity and neuromuscular function [4]. Among the established risk factors, childbirth is the most consistently identified determinant in epidemiological studies, while advancing age and progressive deterioration of pelvic floor support structures further contribute to disease onset [5,6]. The clinical relevance of this condition is underscored by the high proportion of prolapse surgeries performed for recurrent disease, highlighting the need for improved understanding of the determinants influencing surgical outcomes [7,8]. Perioperative outcomes following prolapse surgery remain highly variable, and the capacity to accurately predict surgical morbidity at the individual level is still limited. This heterogeneity reflects the complex interaction between patient characteristics, disease severity, and procedural factors, which are not consistently integrated in current clinical research.

Surgical intervention remains the main therapeutic option for symptomatic prolapse in cases where conservative management is unsuccessful [9]. A range of vaginal reconstructive procedures is currently employed, including anterior and posterior colporrhaphy as well as apical suspension techniques such as sacrospinous ligament fixation [10]. These interventions are intended to re-establish pelvic anatomy and enhance functional outcomes; however, they vary in surgical complexity, extent of tissue dissection, and associated perioperative risk. Despite the diversity of techniques, comparative evidence regarding their impact on perioperative outcomes remains inconsistent, particularly when accounting for patient-specific factors.

Stress urinary incontinence commonly coexists with pelvic organ prolapse, and mid-urethral sling procedures, such as tension-free vaginal tape (TVT) and transobturator tape (TOT), are widely utilized for its surgical treatment [11]. Accordingly, prolapse repair is frequently performed in conjunction with anti-incontinence procedures during the same surgical session. Although this combined approach enables comprehensive management of pelvic floor dysfunction, it may also affect perioperative outcomes, including operative duration, blood loss, postoperative recovery and length of hospital stay [12,13,14]. However, the impact of concomitant anti-incontinence procedures on perioperative outcomes remains inadequately quantified, particularly within heterogeneous surgical populations.

Perioperative outcomes after prolapse surgery exhibit substantial variability across patients and surgical approaches. Beyond procedural factors, elements such as obstetric history, comorbidities, prolapse severity, and prior pelvic surgery may further influence this heterogeneity [10,15]. However, the relative contribution of these factors to perioperative morbidity remains insufficiently characterized, particularly in studies that assess multiple surgical strategies within a single cohort.

In this context, stratified analytical approaches combined with random-effects modeling provide a robust framework for evaluating predictors of perioperative outcomes, enabling integration of data across heterogeneous surgical groups while accounting for inter-group variability. Such methods may enhance the accuracy of effect estimates and generate clinically meaningful insights for surgical decision-making [16,17,18].

Accordingly, this study aimed to systematically evaluate predictors of perioperative outcomes following pelvic organ prolapse surgery using a stratified analytical framework across multiple vaginal reconstructive techniques. The analysis focused on identifying factors associated with intraoperative blood loss, length of hospital stay, postoperative hospital stay and bladder catheterization time, while accounting for variability between surgical procedures.

## 2. Materials and Methods

### 2.1. Study Design, Population and Ethical Considerations

A retrospective cohort study was performed at the County Clinical Emergency Hospital of Sibiu, Romania, including women who underwent surgical treatment for pelvic organ prolapse between January 2020 and December 2025. Eligible cases were identified through screening of the institutional electronic medical records, resulting in a total cohort of 376 patients who met the predefined inclusion criteria.

Patients were stratified according to the reconstructive technique employed. Three surgical approaches were analyzed: sacrospinous ligament fixation with mesh reinforcement combined with a mid-urethral sling (SFM + TOT/TVT), anterior and posterior vaginal repair with concomitant sling (A&P repair + TOT/TVT) and isolated anterior and posterior colporrhaphy without anti-incontinence surgery (A&P repair alone). Sacrocolpopexy was not included in the present analysis because the study was specifically designed to evaluate vaginal reconstructive procedures performed in our institutional cohort. Abdominal, laparoscopic, or robotic sacrocolpopexy represents a different surgical route, with distinct indications, operative anatomy, mesh placement principles and perioperative risk profile; therefore, it was not pooled together with the vaginal procedures analyzed in this study.

Given the retrospective design and the exclusive use of fully anonymized data, this study did not require formal approval from an ethics committee according to applicable institutional and national regulations. The study was conducted in accordance with the principles of the Declaration of Helsinki.

### 2.2. Surgical Procedures

All procedures were carried out by senior urogynecologic surgeons in accordance with standardized departmental protocols. In the SFM + TOT/TVT group, sacrospinous ligament fixation with mesh augmentation was performed in combination with a mid-urethral sling. The second group underwent anterior and posterior colporrhaphy with concurrent sling placement, while the third group included patients managed with isolated anterior and posterior vaginal wall repair.

Mid-urethral sling insertion was performed using either the transobturator tape (TOT) or retropubic tension-free vaginal tape (TVT) technique, based on intraoperative judgment. Key procedural steps included hydrodissection of the vaginal planes, suburethral sling placement using a dedicated introducer system, and sacrospinous fixation was performed using a dedicated sacrospinous ligament fixation device/instrumentation system (AMI, Agency for Medical Innovations GmbH, Feldkirch, Vorarlberg, Austria). Concomitant total hysterectomy was performed when clinically indicated as part of prolapse management in patients with uterine descent and/or when benign uterine pathology or patient-specific surgical considerations supported uterine removal. No hysterectomy was performed for active malignancy, as patients requiring oncologic surgery were excluded from the cohort.

The choice of surgical procedure was not randomized, but was based on clinical examination, prolapse compartment involvement, prolapse severity, uterine status, presence of stress urinary incontinence and surgeon judgment. Sacrospinous fixation was generally selected when apical support was required, whereas anterior and posterior repair was performed for clinically relevant anterior and/or posterior compartment defects. Mid-urethral sling placement was added when stress urinary incontinence was present or clinically indicated.

### 2.3. Eligibility Criteria and Data Collection

Adult women with clinically confirmed pelvic organ prolapse requiring reconstructive surgery were eligible for inclusion, including those presenting with concomitant stress urinary incontinence. Both primary interventions and procedures performed for recurrent prolapse were considered, provided that complete perioperative clinical data were available.

Exclusion criteria comprised incomplete baseline or outcome data, absence of perioperative laboratory investigations, and the performance of additional major pelvic procedures unrelated to prolapse repair during the same operative session. To reduce potential confounding, patients with acute pelvic infection at admission, known immunosuppressive conditions, or active malignancy requiring oncologic surgery were also excluded.

Clinical and perioperative data were extracted from the institutional electronic medical records and included demographic parameters, obstetric history, comorbidities, laboratory findings, surgical characteristics, and postoperative outcomes.

Continuous variables analyzed were age at admission, parity, intraoperative blood loss, total length of hospital stay, postoperative hospitalization duration, and duration of urinary catheterization. Additional variables comprised menopausal status, chronic constipation, smoking status, diabetes mellitus, chronic pulmonary disease, previous gynecologic surgery, and prior hysterectomy.

Prolapse severity was assessed clinically using a four-stage grading system in accordance with established classification criteria. Stress urinary incontinence was categorized as absent, mixed, or pure, while body mass index was used to classify obesity into grades I, II, and III.

Length of hospital stay was defined as the total number of days from admission to discharge. Postoperative hospital stay was defined as the number of days from the day of surgery to hospital discharge. Duration of bladder catheterization was defined as the number of days between catheter placement and catheter removal. The 4-day threshold for postoperative hospital stay was based on the cohort median and was used only for dichotomized statistical analysis, not as a predefined biological marker of recovery. Preoperative imaging data, including pelvic floor ultrasound or magnetic resonance imaging, were not systematically available in the institutional records and were therefore not included in the statistical analysis. Surgical indication and prolapse severity assessment were based primarily on clinical examination and medical record documentation. Difficult delivery was defined as a documented history of prolonged or obstructed labor, instrumental delivery, severe perineal trauma, shoulder dystocia, or any delivery explicitly recorded as difficult in the medical record. Heavy lifting was defined as documented occupational or repetitive physical activity involving regular lifting of heavy loads, as recorded in the patient’s history. Chronic constipation, smoking status, diabetes mellitus, chronic pulmonary disease/chronic cough and menopausal status were extracted from the medical records based on documented clinical history.

### 2.4. Statistical Analysis

Continuous variables were described as means with standard deviations, while categorical variables were presented as frequencies and percentages. Comparisons between groups were performed using one-way analysis of variance (ANOVA) for continuous data and either the chi-square test or Fisher’s exact test for categorical variables, as appropriate. When overall between-group comparisons were statistically significant, post hoc pairwise analyses were performed. For continuous variables, Tukey’s honestly significant difference test was used after ANOVA. For categorical variables, pairwise chi-square or Fisher’s exact tests with Bonferroni correction were applied, as appropriate.

Associations between potential predictors and perioperative outcomes—including intraoperative blood loss, length of hospital stay, postoperative hospital stay and bladder catheterization time—were assessed separately within each surgical group. Odds ratios with 95% confidence intervals were calculated for each stratum and subsequently pooled using a random-effects model based on the DerSimonian–Laird method, allowing for between-group variability. Statistical heterogeneity was evaluated using Cochran’s Q test and quantified by the I^2^ statistic.

All analyses were conducted using a two-sided significance threshold of 0.05. Data processing and statistical modeling were performed in Python version 3.12 (Python Software Foundation) using the PyCharm development environment version 2025.3.2.1 (JetBrains). For variables showing moderate or high heterogeneity across surgical strata, particularly when I^2^ exceeded 50% and Cochran’s Q test was significant, pooled random-effects estimates were interpreted as exploratory summary measures only. In these cases, clinical interpretation was based primarily on the presence of between-group variability rather than on the pooled odds ratio alone. Therefore, heterogeneous pooled estimates were not used to support direct procedure-independent clinical recommendations.

## 3. Results

The study cohort comprised 376 patients allocated to three surgical groups: SFM + TOT/TVT (n = 83), A&P repair + TOT/TVT (n = 180), and A&P repair alone (n = 113). Patients in the A&P repair group exhibited significantly longer total hospitalization, postoperative stay, and duration of bladder catheterization compared with the other groups (all *p* < 0.001) (Table 1).

Intraoperative blood loss varied significantly across groups, with the highest values observed in the A&P repair group (*p* = 0.001). Admission hemoglobin levels differed significantly (*p* = 0.007), whereas no significant differences were found in discharge hemoglobin values (*p* = 0.077) (Table 1).

Concomitant total hysterectomy was performed more frequently in the A&P repair group (49.6%) compared with the A&P repair + TOT/TVT group (20.0%) and the SFM + TOT/TVT group (10.8%) (*p* < 0.001). The incidence of postoperative complications was comparable between groups (9.6% vs. 8.9% vs. 7.1%, *p* = 0.792).

Stratified analysis using a random-effects model (DerSimonian–Laird) identified multiple factors significantly associated with intraoperative blood loss exceeding the cohort median (350 mL) (Table 2). Among surgical history variables, concomitant total hysterectomy showed the largest unadjusted association with intraoperative blood loss above the cohort median (OR = 65.3, 95% CI [30.0–142]). However, the magnitude of this estimate should be interpreted cautiously, as concomitant hysterectomy was unevenly distributed across surgical groups and may reflect associated surgical complexity, prolapse severity, and surgeon selection rather than an isolated causal effect. Prior hysterectomy was associated with a reduced risk of excessive bleeding (OR = 0.30, 95% CI [0.11–0.81]). Previous gynecological surgery did not reach statistical significance (OR = 0.47, 95% CI [0.22–1.01]).

Regarding obstetric history, delivery of neonates weighing over 4 kg (OR = 2.19, 95% CI [1.25–3.86]) and forceps-assisted delivery (OR = 2.84, 95% CI [1.08–7.48]) were significantly associated with increased bleeding risk. Other variables, including difficult delivery, multiple pregnancies, and parity above the cohort median, were not statistically significant, although parity exhibited moderate heterogeneity (I^2^ = 55.9%).

Among comorbidities, chronic constipation was significantly associated with increased blood loss (OR = 2.16, 95% CI [1.33–3.51]), while menopause, smoking, chronic pulmonary disease or cough, intense physical activity, and diabetes mellitus showed no significant associations.

Prolapse severity demonstrated a graded relationship with bleeding risk, with higher stages associated with increased odds ratios. Grade II obesity was significantly associated with increased intraoperative bleeding (OR = 2.75, 95% CI [1.39–5.43]), whereas grades I and III did not reach statistical significance, although grade I showed moderate heterogeneity (I^2^ = 65.8%). Age above the cohort median (63 years) was also significantly associated with increased bleeding risk (OR = 1.82, 95% CI [1.16–2.87]), while catheterization duration was not significantly related to this outcome.

Overall, most variables exhibited low heterogeneity (I^2^), indicating consistent effects across surgical groups. Moderate to high heterogeneity was observed for grade II prolapse (I^2^ = 74.0%), grade III prolapse (I^2^ = 65.2%), grade I obesity (I^2^ = 65.8%), and parity (I^2^ = 55.9%). For grade II prolapse, Cochran’s Q test was statistically significant (p_het = 0.0215), suggesting true between-group variability and warranting cautious interpretation of pooled estimates. Because significant heterogeneity was observed for grade II prolapse, the pooled estimate for this variable should be considered exploratory. The elevated pooled odds ratio indicates a possible association with increased intraoperative bleeding, but the magnitude and direction of the effect may differ across surgical strata. Therefore, this result should not be interpreted as a uniform effect applicable to all procedures. Table 2 summarizes exploratory stratified associations between clinical predictors and intraoperative blood loss above the cohort median. The purpose of this table is to identify variables that may act as markers of increased operative complexity rather than to provide a definitive prediction model.

Stratified random-effects analysis (DerSimonian–Laird) identified multiple factors associated with hospitalization exceeding the cohort median (7 days) (Table 3). Concomitant total hysterectomy was significantly linked to prolonged stay (OR = 2.05, 95% CI [1.00–4.20]), with moderate between-group heterogeneity (I^2^ = 43.4%), whereas previous hysterectomy, prior gynecologic surgery and postoperative complications were not significant predictors.

Among obstetric variables, delivery of neonates > 4 kg (OR = 2.52, 95% CI [1.37–4.62]) and difficult deliveries (OR = 1.91, 95% CI [1.21–3.00]) were significantly associated with extended hospitalization. Parity ≥ 3 was also significant (OR = 1.73, 95% CI [1.04–2.86]), while forceps-assisted delivery and multiple pregnancies were not.

No significant associations were identified for comorbidities or stress urinary incontinence categories. Regarding prolapse severity, grade II was significantly associated with prolonged hospitalization (OR = 1.90, 95% CI [1.04–3.47]). Grade III showed an increased effect (OR = 3.45) but did not reach statistical significance and exhibited moderate-to-high heterogeneity (I^2^ = 68.3%, p_het = 0.0426), requiring cautious interpretation. Grades I and IV were not associated with this outcome. Given the significant between-group heterogeneity, the pooled estimate for grade III prolapse was not considered sufficiently stable to support a procedure-independent conclusion. This finding suggests that the relationship between grade III prolapse and prolonged hospitalization may be influenced by the type of surgical procedure and should therefore be interpreted at the stratum level.

Increased intraoperative blood loss above the cohort median (>350 mL) was significantly associated with prolonged hospitalization (OR = 1.65, 95% CI [1.03–2.63]). This association was interpreted as a general marker of increased operative burden and postoperative monitoring requirements rather than as a POP-specific causal mechanism. Overall, heterogeneity was low for most variables, supporting the consistency of effect estimates across surgical groups, except for grade III prolapse, which showed significant variability. Table 3 presents predictors associated with total hospital stay above the cohort median. The clinically relevant findings are those related to concomitant hysterectomy, obstetric burden, prolapse severity, and increased intraoperative bleeding.

Stratified random-effects analysis (DerSimonian–Laird) identified a limited number of factors associated with postoperative stay exceeding the cohort median (4 days), with greater heterogeneity observed compared to previous analyses (Table 4).

None of the surgical history variables reached statistical significance, including concomitant total hysterectomy, previous hysterectomy, prior gynecologic surgery, and postoperative complications, although concomitant hysterectomy approached significance (OR = 1.67, 95% CI [0.93–2.98]).

Among obstetric variables, delivery of neonates > 4 kg was significantly associated with prolonged postoperative stay (OR = 1.89, 95% CI [1.05–3.41]). Difficult deliveries showed a modest effect (OR = 1.30) but were accompanied by moderate-to-high heterogeneity (I^2^ = 69.0%, p_het = 0.0398), limiting interpretability. Forceps-assisted delivery, multiple pregnancies, and parity were not significant.

No significant associations were identified for comorbidities or stress urinary incontinence categories. Regarding prolapse severity, grade IV prolapse was significantly associated with prolonged postoperative stay (OR = 2.98, 95% CI [1.04–8.51]) without heterogeneity, indicating consistent effects across groups. Grade III prolapse showed a high odds ratio (OR = 3.31) but with very wide confidence intervals and substantial heterogeneity (I^2^ = 84.8%, p_het = 0.0014), limiting its reliability. Grades I and II were not significant.

Obesity categories were not significantly associated with postoperative duration, although grade II showed a trend (OR = 1.70, 95% CI [0.91–3.17]). None of the dichotomized continuous variables reached statistical significance. Age above the cohort median showed a modest effect (OR = 1.48) with moderate heterogeneity (I^2^ = 67.0%, p_het = 0.0483), while intraoperative blood loss and parity also showed non-significant trends (OR ≈ 1.60).

Overall, heterogeneity was more pronounced in this analysis, particularly for grade III prolapse and age, indicating variability in effect estimates across surgical groups and requiring cautious interpretation of pooled results. Table 4 evaluates factors associated with postoperative hospital stay above the cohort median. Because several variables showed relevant heterogeneity, the results should be interpreted as exploratory and context-dependent.

Stratified analysis using a random-effects model (DerSimonian–Laird) identified a limited number of factors significantly associated with bladder catheterization duration exceeding the cohort median (3 days). Heterogeneity represented an important limitation for several variables included in the analysis (Table 5).

Among surgical history variables, postoperative complications showed the strongest association with prolonged bladder catheterization (OR = 6.33, 95% CI [2.85–14.0]). This effect was observed without heterogeneity, indicating high consistency across the surgical groups (SFM + TOT/TVT, A&P repair + TOT/TVT, and A&P repair alone). In contrast, concomitant total hysterectomy, previous hysterectomy, and prior gynecologic surgery were not significantly associated with prolonged catheterization.

Among obstetric history variables, none reached statistical significance. Delivery of neonates weighing more than 4 kg showed a suggestive association (OR = 2.18, 95% CI [0.98–4.89]), approaching the threshold of significance. Difficult deliveries overall yielded an OR of 1.80 but were accompanied by moderate-to-high heterogeneity (I^2^ = 72.3%, p_het = 0.0272), limiting the interpretability of the pooled estimate. In contrast, parity above the cohort median was significantly associated with prolonged bladder catheterization (OR = 2.52, 95% CI [1.35–4.69]) with low heterogeneity (I^2^ = 5.2%), supporting higher parity as a consistent predictor of extended postoperative catheterization.

Among comorbidities and risk factors, none of the evaluated variables reached statistical significance. Intense physical exertion showed moderate heterogeneity (I^2^ = 57.7%) but no significant association. Menopause, chronic obstructive pulmonary disease or chronic cough, and diabetes mellitus yielded non-significant odds ratios with no detectable heterogeneity. Similarly, stress urinary incontinence categories were not significantly associated with catheterization duration; both categories showed subunitary odds ratios (OR ≈ 0.28) without reaching statistical significance.

In the analysis of pelvic organ prolapse severity, grade I prolapse was significantly associated with prolonged bladder catheterization (OR = 4.66, 95% CI [1.84–11.8]) with no observed heterogeneity, indicating strong consistency across groups and representing a robust finding. Grade III prolapse showed an elevated OR (2.46) but with moderate-to-high heterogeneity (I^2^ = 71.2%, p_het = 0.0310), requiring cautious interpretation. Grade II and grade IV prolapse were not significantly associated with catheterization duration.

Regarding obesity, none of the obesity categories showed significant associations with catheterization duration. The I^2^ values were negligible across all obesity grades, indicating the absence of a consistent effect of body mass index on this outcome.

Among the dichotomized continuous variables, age above the cohort median showed an OR of 2.11 but was accompanied by high heterogeneity (I^2^ = 74.7%, p_het = 0.0193), making the pooled estimate unreliable for interpretation. Intraoperative blood loss above the median was not significantly associated with prolonged bladder catheterization (OR = 1.03).

Overall, heterogeneity was a notable feature of this analysis. Moderate or high I^2^ values were observed for difficult deliveries (I^2^ = 72.3%), grade III prolapse (I^2^ = 71.2%), age at admission (I^2^ = 74.7%), and intense physical exertion (I^2^ = 57.7%). For these variables, Cochran’s Q test reached statistical significance, indicating genuine variability in effect estimates between the surgical groups and limiting the interpretability of the corresponding pooled odds ratios. Table 5 evaluates factors associated with prolonged bladder catheterization. Clinically meaningful findings include postoperative complications and parity, while heterogeneous estimates should be interpreted cautiously.

Multiple linear regression analysis evaluated the contribution of surgical procedure type as a predictor of the four main perioperative outcomes, using SFM + TOT/TVT as the reference category. The results of the four regression models are presented within the same table (Table 6).

Model 1—Length of hospital stay (R^2^ = 0.062, adjusted R^2^ = 0.057, F = 12.306, *p* < 0.0001) identified surgical procedure type as a significant predictor of hospitalization duration, accounting for 6.2% of the total variance. Compared with the reference group (SFM + TOT/TVT), patients undergoing A&P repair + TOT/TVT had a mean increase of 0.66 days in hospital stay (β = 0.655, 95% CI [0.011–1.300], *p* = 0.0464, standardized β = 0.129), indicating a modest but statistically significant effect of combining colporrhaphy with sling placement.

A stronger effect was observed for the A&P repair alone group, which was associated with an increase of 1.71 hospitalization days compared to the reference group (β = 1.711, 95% CI [1.008–2.413], *p* < 0.0001, standardized β = 0.309), suggesting a substantial independent impact on hospital stay duration.

**Figure 1:**
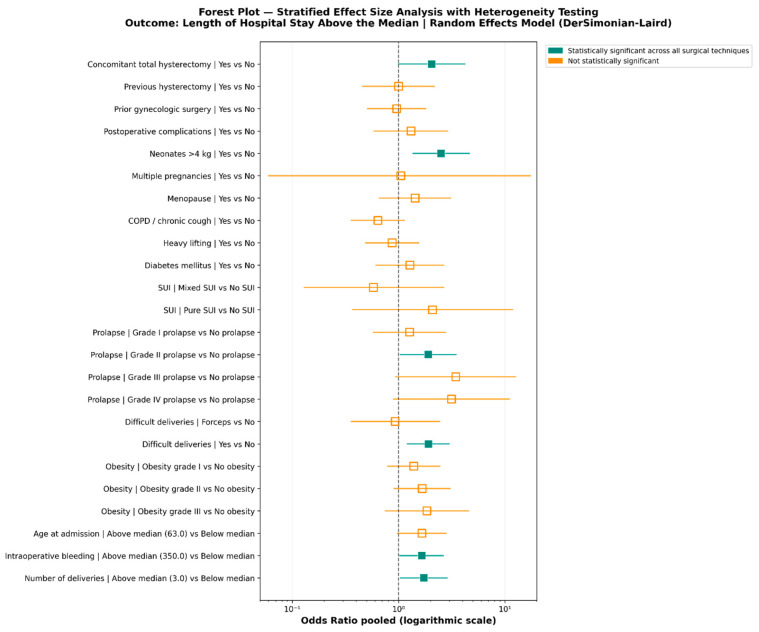
Forest plot of stratified effect size analysis for length of hospital stay above the median.

Despite statistical significance, the relatively low explanatory power of the model (R^2^ = 6.2%) indicates that surgical procedure type accounts for only a limited proportion of variability, with the remaining variance likely influenced by patient-related factors such as comorbidities, postoperative complications, prolapse severity, and age, as highlighted in the stratified analyses (Table 3 and Table 6) (Figure 1).

Model 2—Postoperative duration (R^2^ = 0.080, adjusted R^2^ = 0.075, F = 16.229, *p* < 0.0001) demonstrated the highest explanatory capacity among the models, with surgical procedure type accounting for 8.0% of the variance in postoperative recovery time. The adjusted R^2^ confirms the stability of the model. Compared with the reference group (SFM + TOT/TVT), patients in the A&P repair + TOT/TVT group experienced a 0.69-day increase in postoperative duration (β = 0.690, 95% CI [0.206–1.175], *p* = 0.0054, standardized β = 0.179), indicating a significant effect of increased procedural complexity on early recovery.

**Figure 2:**
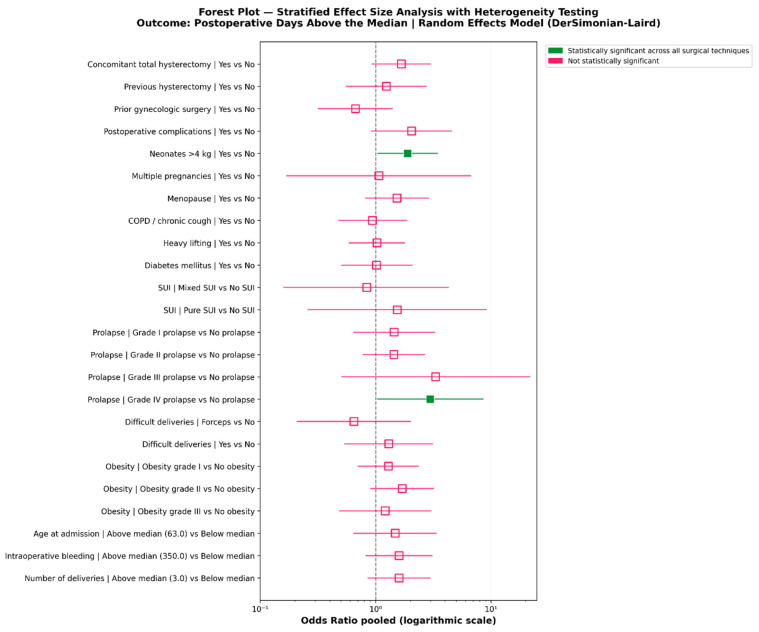
Forest plot of stratified effect size analysis for postoperative days above the median.

The A&P repair alone group showed the greatest effect, with an average increase of 1.51 postoperative days compared with the reference group (β = 1.509, 95% CI [0.981–2.037], *p* < 0.0001, standardized β = 0.359). This represents the highest standardized coefficient across all models, highlighting the strong independent influence of this procedure on immediate postoperative recovery. The narrow confidence interval further supports the precision of this estimate (Table 6) (Figure 2).

Model 3—Duration of bladder catheterization (R^2^ = 0.059, adjusted R^2^ = 0.054, F = 11.745, *p* < 0.0001) identified procedure type as a significant predictor, accounting for 5.9% of the variance in catheterization duration. Compared with the reference group (SFM + TOT/TVT), patients undergoing A&P repair + TOT/TVT required an average of 0.54 additional catheterization days (β = 0.536, 95% CI [0.169–0.903], *p* = 0.0043, standardized β = 0.185), indicating a significant effect of increased reconstructive extent on postoperative bladder function.

**Figure 3:**
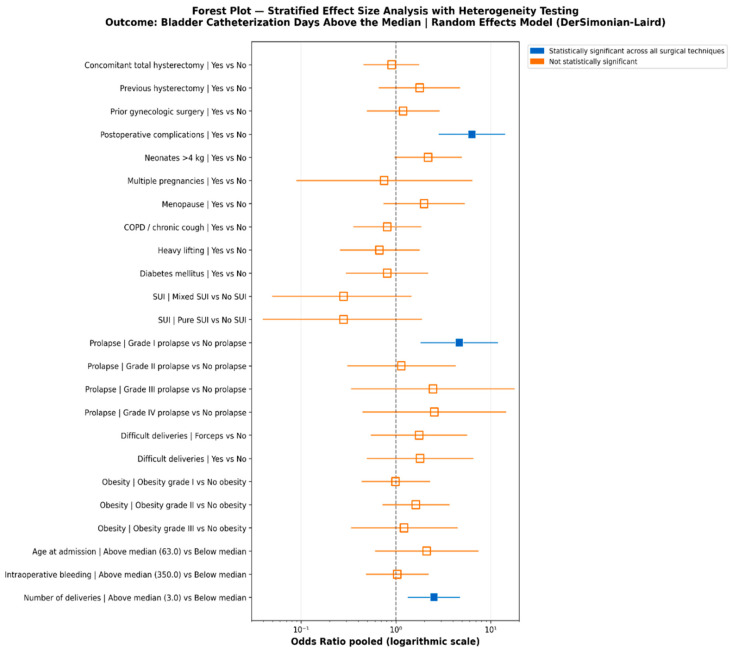
Forest plot of stratified effect size analysis for bladder catheterization days above the median.

The A&P repair alone group exhibited the greatest increase, with an average prolongation of 0.98 days compared to the reference group (β = 0.984, 95% CI [0.585–1.384], *p* < 0.0001, standardized β = 0.313). The narrow confidence interval supports the precision of this estimate. Although the absolute difference is modest, it is consistent with the gradient of surgical complexity observed across outcomes and reflects the cumulative impact of more extensive pelvic reconstruction on bladder recovery (Table 6) (Figure 3).

Model 4—Intraoperative blood loss (R^2^ = 0.035, adjusted R^2^ = 0.030, F = 6.820, *p* = 0.0012) showed the lowest explanatory capacity, with procedure type accounting for only 3.5% of the variance in intraoperative bleeding. The minimal difference between R^2^ and adjusted R^2^ indicates slight overestimation, consistent with the limited number of predictors. Unlike the previous models, no comparison reached statistical significance relative to the reference group (SFM + TOT/TVT).

The A&P repair + TOT/TVT group demonstrated a non-significant reduction in blood loss (β = −14.455, 95% CI [−35.853–6.944], *p* = 0.1849, standardized β = −0.087), while the A&P repair alone group showed a non-significant increase (β = 21.900, 95% CI [−1.415–45.215], *p* = 0.0655, standardized β = 0.121), with confidence intervals crossing zero in both cases.

**Figure 4:**
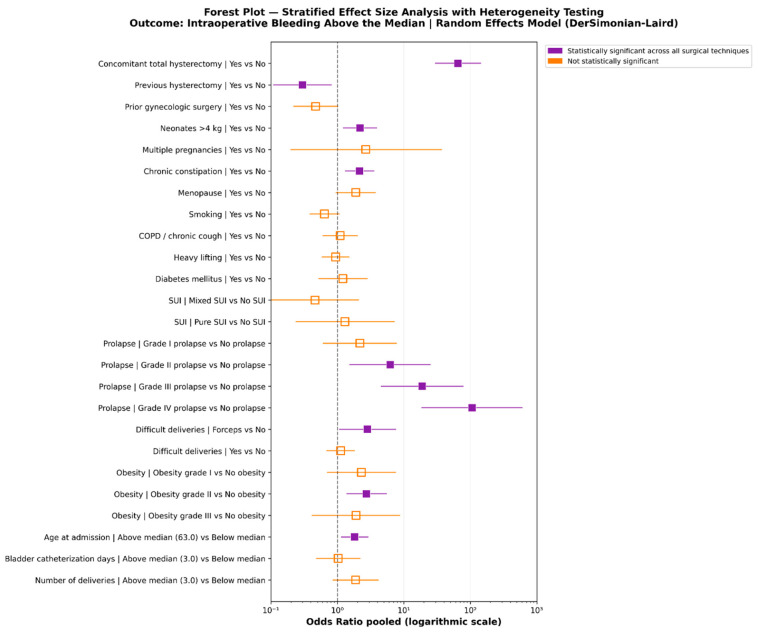
Forest plot of stratified effect size analysis for intraoperative bleeding above the median.

Overall, the lack of statistical significance and low explanatory power indicate that procedure type alone is a weak predictor of intraoperative bleeding, which appears to be primarily influenced by patient-related factors such as prolapse severity, concomitant hysterectomy, obesity, and age, as demonstrated in the stratified analysis (Table 6) (Figure 4).

## 4. Discussion

The present study offers an integrated assessment of perioperative outcomes following pelvic organ prolapse (POP) surgery across multiple vaginal reconstructive approaches, highlighting that early postoperative morbidity arises from the combined influence of procedural characteristics and patient-specific factors. A key strength of this analysis lies in its evaluation of predictors within a heterogeneous surgical cohort, rather than restricting the investigation to a single operative technique. This approach enhances clinical relevance, as POP surgery is frequently characterized by varied procedural combinations, the inclusion of concomitant continence interventions, and differences in baseline pelvic floor impairment, all of which complicate direct comparisons of perioperative outcomes. The existing literature similarly underscores the substantial heterogeneity inherent in prolapse surgery populations and reported outcomes [16,19]. However, when substantial heterogeneity was detected, pooled random-effects estimates were not interpreted as evidence of a uniform association across all procedures. In particular, variables such as grade II and grade III prolapse showed relevant between-group variability, indicating that their effects may depend on the surgical context. For these variables, the pooled odds ratios should be viewed as exploratory summaries, while clinical interpretation should rely on procedure-specific patterns rather than on a single overall estimate.

The most consistent observation in this cohort was the significant impact of surgical approach on length of hospital stay, postoperative recovery and duration of bladder catheterization. Patients undergoing isolated anterior and posterior repair exhibited less favorable short-term recovery profiles compared with those treated with sacrospinous fixation combined with a sling procedure. This pattern aligns with prior comparative evidence indicating that surgical route, extent of reconstruction, and the use of concomitant procedures influence early postoperative burden, even in the context of similar long-term anatomical outcomes. This concept is supported by findings from the OPTIMAL trial and corroborated by more recent evidence syntheses addressing uterine management in prolapse surgery [20].

Importantly, procedure type in our regression models was more clearly associated with recovery-related outcomes than with intraoperative blood loss. This distinction is clinically plausible. Intraoperative bleeding in prolapse surgery is unlikely to depend only on the procedural label; it is also shaped by distorted anatomy, associated hysterectomy, tissue vascularity, prior pelvic surgery, and prolapse severity. That interpretation is supported by the literature showing that perioperative morbidity in POP surgery is multifactorial rather than purely technique-driven [19,21].

One of the most important findings in the present study was the strong association between concomitant total hysterectomy and increased intraoperative blood loss. The direction of this effect is well supported by comparative evidence. The best available syntheses show that uterine-preserving prolapse procedures are generally associated with lower blood loss and shorter operative time than prolapse surgery combined with hysterectomy [20,22]. Accordingly, although the magnitude of the odds ratio observed in our cohort is unusually high and should be interpreted cautiously, the biological and surgical direction of the association is credible. The unusually high magnitude of the association between concomitant hysterectomy and increased intraoperative blood loss most likely reflects the combined effect of additional surgical steps, more advanced pelvic floor pathology, and non-random surgical selection. Therefore, this estimate should not be interpreted as a precise independent effect size comparable to adjusted estimates reported in previous studies. Rather, it identifies concomitant hysterectomy as a marker of increased operative complexity within this cohort.

Conversely, a history of prior hysterectomy was associated with a reduced risk of intraoperative bleeding in the present analysis. Although this observation should be interpreted with caution, it is biologically plausible. In patients with prior hysterectomy, reconstructive procedures may involve less extensive dissection of uterine vascular structures and fewer additional surgical steps, potentially limiting intraoperative blood loss in selected cases. This interpretation is consistent with comparative evidence examining uterine-preserving versus hysterectomy-based approaches in prolapse surgery [22,23].

The indication for concomitant hysterectomy is clinically relevant when interpreting perioperative outcomes. In the present cohort, hysterectomy should not be interpreted simply as an additional binary procedure, but as a marker of a more complex surgical context. In some patients, it was related to uterine descent as part of prolapse treatment, whereas in others it reflected associated benign uterine pathology or individualized surgical decision-making. This distinction is important because contemporary prolapse surgery increasingly considers uterine-preserving approaches when appropriate, while hysterectomy may increase operative dissection, vascular exposure and postoperative burden.

Obstetric history also emerged as clinically relevant in our study, particularly the delivery of neonates weighing more than 4 kg and forceps-assisted birth. These findings are biologically plausible because long-term pelvic floor trauma after childbirth is strongly linked to levator ani injury and later prolapse-related dysfunction. Recent high-level evidence supports this mechanism. Forceps delivery was identified as a major risk factor for levator ani avulsion, and the same review also highlighted other obstetric predictors of structural pelvic floor trauma [24]. In parallel, the lasting clinical consequences of levator injury on pelvic floor dysfunction were underlined [25]. Although those papers do not study intraoperative bleeding specifically, they strongly support the mechanistic interpretation that prior obstetric trauma may worsen tissue support, alter anatomy, and complicate later reconstructive surgery.

The association between prolapse severity and adverse perioperative outcomes observed in this cohort is clinically consistent. Advanced stages are typically characterized by greater anatomical distortion, more technically demanding dissection, and increased surgical complexity. However, the very high odds ratios identified in certain strata, particularly for advanced prolapse, should not be interpreted as precise quantitative estimates. Instead, they should be viewed as indicators of a strong adverse relationship, potentially influenced by sparse data and limited subgroup sizes. This cautious interpretation is supported by existing evidence linking severe or recurrent pelvic floor pathology with compromised structural support and increased procedural complexity [25,26,27].

Our data also suggest that prolonged postoperative recovery is influenced by a combination of surgical burden and perioperative events. Our data also suggest that prolonged hospitalization is influenced by a combination of surgical burden and perioperative events. The association between increased intraoperative blood loss and longer hospitalization should not be interpreted as a finding specific to pelvic organ prolapse surgery, as this relationship is expected across many surgical fields. In the present cohort, however, intraoperative blood loss remains clinically relevant because it may reflect greater operative complexity, more difficult dissection, distorted pelvic anatomy, advanced prolapse severity, or the additional surgical burden of concomitant hysterectomy. Therefore, blood loss was interpreted as a marker of perioperative complexity and postoperative monitoring needs, rather than as an independent POP-specific mechanism. Similarly, the signal observed for concomitant hysterectomy and higher parity suggests that cumulative pelvic floor damage and more extensive surgery can both contribute to slower functional recovery [19,28].

Technique-specific hemorrhagic risks should also be considered when interpreting intraoperative blood loss. Sacrospinous ligament fixation is performed in close anatomical proximity to relevant pelvic vessels, including branches of the internal pudendal and inferior gluteal vascular systems. Although severe hemorrhagic complications are uncommon, vascular injury during sacrospinous fixation has been reported and may require additional hemostatic measures, surgical packing, vascular control, or arterial embolization. This context supports the interpretation of intraoperative bleeding as a procedure- and anatomy-dependent event rather than as a uniform outcome explained only by the broad surgical category.

Postoperative bladder catheterization represents a clinically relevant endpoint, reflecting early lower urinary tract recovery while directly influencing patient comfort and discharge planning. In the present cohort, prolonged catheterization was associated with postoperative complications and higher parity, with additional variability attributable to procedural differences. These findings are consistent with existing evidence highlighting the clinical relevance of postoperative voiding dysfunction after prolapse repair, where transient urinary retention is common and the use of concomitant mid-urethral slings, as well as specific surgical combinations, increases the likelihood of discharge with indwelling catheterization [29,30]. The finding that parity was associated with longer catheterization is also plausible from a pathophysiological perspective. Repeated childbirth may lead to cumulative neuromuscular pelvic floor damage, impaired urethral support, and altered postoperative voiding adaptation. Although the precise endpoint of catheterization duration is not uniformly reported across studies, the broader relationship between childbirth-related pelvic floor trauma and later dysfunction is consistent [24,25,26]. Some findings require cautious interpretation. For example, previous hysterectomy was not associated with prolonged bladder catheterization, despite the possibility of altered pelvic anatomy after prior surgery. This may be explained by standardized postoperative bladder management protocols, the dominance of immediate postoperative complications over remote surgical history, or limited statistical power within this subgroup. Alternatively, prior hysterectomy may represent a heterogeneous clinical category, including patients with different indications, time intervals since surgery, and anatomical conditions. Therefore, this negative association should not be interpreted as evidence that previous hysterectomy has no anatomical relevance, but rather as an absence of detectable association in this cohort.

A major strength of the present study lies in the use of a stratified analytical framework combined with random-effects pooling across clinically distinct surgical groups. In a field in which available evidence often either merges heterogeneous procedures into broad analytical categories or focuses narrowly on a single surgical technique, this approach allows a more structured evaluation of whether associations remain consistent across procedure-specific strata. Nevertheless, several methodological limitations must be acknowledged. First, the retrospective design entails an inherent risk of selection bias, residual confounding, and surgeon-dependent decision-making, all of which may influence both procedure selection and perioperative outcomes. Second, although dichotomizing continuous outcomes may improve clinical interpretability, it also reduces statistical information and may contribute to unstable or inflated effect estimates, particularly in sparse subgroups. These concerns are especially relevant for very large odds ratios and for pooled estimates affected by substantial between-group heterogeneity. Accordingly, such findings should be interpreted as exploratory signals rather than definitive procedure-independent predictors. Future prospective studies using standardized outcome definitions, adjusted modeling strategies, and more balanced surgical strata are required to validate these observations and refine perioperative risk assessment in prolapse surgery [20,26]. Several additional limitations should be acknowledged. Very large odds ratios, particularly the estimate observed for concomitant hysterectomy, may have been influenced by sparse-data effects, imbalance between surgical subgroups and residual confounding. Consequently, these estimates should be interpreted as indicators of potential clinical relevance rather than as precise measures of independent effect. Future prospective studies using adjusted multivariable models, or penalized regression approaches in the presence of sparse data, are required to generate more stable and reliable estimates. Moreover, although intraoperative blood loss was analyzed as a quantitative perioperative outcome, the retrospective nature of the study did not allow a detailed reconstruction of the exact bleeding source, the anatomical site involved, or the occurrence of vessel-specific complications. Another relevant limitation is the absence of systematically collected preoperative imaging data. Contemporary evaluation of pelvic organ prolapse may benefit from pelvic floor ultrasound or magnetic resonance imaging, particularly for anatomical characterization, identification of levator ani defects, assessment of hiatal ballooning and preoperative surgical planning. Because such imaging data were not consistently available in the present retrospective cohort, their potential contribution to perioperative outcome prediction could not be assessed [31]. Procedure selection was indication-driven rather than randomized. Therefore, some surgical techniques may have been preferentially used in patients with specific prolapse patterns or greater anatomical complexity. This represents a potential source of selection bias and should be considered when interpreting differences between surgical groups.

The absence of sacrocolpopexy represents an important boundary of the present study. Sacrocolpopexy is a relevant contemporary procedure for apical prolapse repair, particularly in abdominal, laparoscopic, or robotic approaches. However, because the present cohort focused on vaginal reconstructive techniques, the findings should not be extrapolated to sacrocolpopexy. Future comparative studies including both vaginal and abdominal or minimally invasive apical suspension techniques are needed to better characterize procedure-specific perioperative risk.

From a clinical standpoint, these findings support the need for individualized perioperative risk assessment in women undergoing prolapse surgery. Factors such as concomitant hysterectomy, obstetric history, prolapse severity and postoperative complications appear to play a role comparable to, or greater than, the surgical technique itself in determining early morbidity. In practical terms, surgical planning should extend beyond anatomical correction to include anticipation of recovery burden and the risk of postoperative voiding dysfunction [16,22,29].

## 5. Conclusions

Perioperative outcomes after pelvic organ prolapse surgery are not determined by procedure type alone, but by a complex interplay between surgical approach, disease severity, and patient-specific characteristics. While surgical technique was significantly associated with postoperative recovery-related outcomes, the low explanatory power of the regression models indicates that procedure type alone has limited predictive utility for individualized risk stratification. Recovery after POP surgery is therefore more appropriately understood as the result of combined procedural, anatomical, and patient-specific factors.

In contrast, intraoperative bleeding is predominantly driven by clinical and anatomical factors, particularly prolapse severity and the need for concomitant hysterectomy, emphasizing that surgical context outweighs procedural choice in predicting intraoperative risk. Obstetric history, notably macrosomia and high parity, further amplifies perioperative vulnerability, reflecting the lasting impact of pelvic floor trauma.

The stratified analytical framework employed in this study enables a more rigorous evaluation of predictors across heterogeneous surgical settings and supports a more nuanced exploratory assessment of perioperative risk, but requires validation in adjusted prospective models before being used for individualized prediction. Collectively, these findings argue for a shift toward individualized surgical planning that integrates both patient-related and procedural determinants. Future prospective investigations are required to validate these observations and to support the development of predictive models for optimized patient selection and perioperative management.

## Figures and Tables

**Table 1:** Perioperative data by surgical procedure groups.

Variable	SFM + TVT/TOT (n = 83)	A&P Repair + TVT/TOT (n = 180)	A&P Repair Alone (n = 113)	*p*-Value
Perioperative Data—Continuous (Mean ± SD)				
Length of hospital stay (days)	6.29 ± 3.05	6.94 ± 2.20	8.00 ± 2.42	<0.001
Postoperative stay (days)	3.48 ± 2.14	4.17 ± 1.69	4.99 ± 1.90	<0.001
Duration of bladder catheterization (days)	2.33 ± 1.16	2.86 ± 1.41	3.31 ± 1.55	<0.001
Intraoperative blood loss (mL)	360.84 ± 78.88	346.39 ± 71.31	382.74 ± 98.60	0.001
Hemoglobin at admission (g/dL)	13.25 ± 1.31	13.69 ± 1.04	13.44 ± 1.07	0.007
Hemoglobin at discharge (g/dL)	11.62 ± 1.36	11.80 ± 1.16	11.47 ± 1.24	0.077
Perioperative Data—Categorical N (%)				
Concomitant total hysterectomy	9 (10.8%)	36 (20.0%)	56 (49.6%)	<0.001
Postoperative complications	8 (9.6%)	16 (8.9%)	8 (7.1%)	0.792

**Table 2:** Stratified analysis of effect size—intraoperative bleeding above the median. *Random Effects Model (DerSimonian–Laird)*.

Variable	Comparison	OR	95% CI	I^2^%	Q	p_het	k	Sign.
Surgical history								
Concomitant total hysterectomy	Yes vs. No	65.3	[30.0–142]	0.0%	0.84	0.6572	3	*
Previous hysterectomy	Yes vs. No	0.30	[0.11–0.81]	0.0%	0.34	0.8416	3	*
Prior gynecologic surgery	Yes vs. No	0.47	[0.22–1.01]	2.0%	2.04	0.3603	3	
Obstetric history								
Neonates > 4 kg	Yes vs. No	2.19	[1.25–3.86]	0.0%	1.50	0.4731	3	*
Multiple pregnancies	Yes vs. No	2.68	[0.20–36.6]	34.1%	1.52	0.2180	2	
Difficult deliveries								
	Forceps vs. No	2.84	[1.08–7.48]	0.0%	0.00	0.9992	3	*
	Yes vs. No	1.12	[0.70–1.79]	0.0%	0.68	0.7129	3	
Number of deliveries	Above median (3.0) vs. Below median	1.88	[0.86–4.09]	55.9% *	4.54	0.1036	3	
Chronic constipation	Yes vs. No	2.16	[1.33–3.51]	0.0%	1.59	0.4525	3	*
Menopause	Yes vs. No	1.89	[0.96–3.70]	0.0%	0.26	0.8781	3	
Smoking	Yes vs. No	0.64	[0.39–1.06]	0.0%	0.15	0.9261	3	
COPD/chronic cough	Yes vs. No	1.10	[0.61–1.98]	0.0%	0.92	0.6321	3	
Heavy lifting	Yes vs. No	0.94	[0.59–1.49]	0.0%	1.34	0.5121	3	
Diabetes mellitus	Yes vs. No	1.21	[0.53–2.80]	18.7%	2.46	0.2922	3	
Stress urinary incontinence (SUI)								
SUI								
Mixed SUI vs. No SUI		0.46	[0.10–2.08]	0.0%	0.40	0.8199	3	
Pure SUI vs. No SUI		1.30	[0.24–7.14]	0.0%	0.37	0.8293	3	
Associated compartment prolapse severity								
Grade I associated prolapse vs. absence of associated prolapse in that compartment		2.18	[0.62–7.69]	45.6%	3.68	0.1590	3	
Grade II prolapse vs. No prolapse		6.23	[1.55–24.9]	74.0% *	7.68	0.0215	3	*
Grade III prolapse vs. No prolapse		18.9	[4.63–77.5]	65.2% *	5.75	0.0564	3	*
Grade IV prolapse vs. No prolapse		106.3	[18.8–600]	0.0%	0.20	0.9070	3	*
Obesity								
Obesity grade I vs. No obesity		2.30	[0.71–7.44]	65.8% *	5.84	0.0538	3	
Obesity grade II vs. No obesity		2.75	[1.39–5.43]	0.0%	0.68	0.7101	3	*
Obesity grade III vs. No obesity		1.91	[0.42–8.62]	25.5%	2.68	0.2614	3	
Age at admission	Above median (63.0) vs. Below median	1.82	[1.16–2.87]	0.0%	0.61	0.7370	3	*
Bladder catheterization days	Above median (3.0) vs. Below median	1.03	[0.49–2.18]	33.1%	2.99	0.2242	3	

OR = pooled Odds Ratio|95% CI = 95% confidence interval|I^2^ = heterogeneity. I^2^ > 50% = moderate heterogeneity|I^2^ > 75% = high heterogeneity. p_het = *p*-value of Cochran’s Q test|* = 95% CI does not include 1 (statistically significant). Variables with I^2^ > 50% and significant Cochran’s Q test were considered heterogeneous; their pooled estimates should be interpreted as exploratory and not as uniform effects across all surgical groups. Colors: yellow = statistically significant association (OR with 95% CI excluding the null value). The term “no prolapse” refers to the absence of associated prolapse in the evaluated compartment, not to the absence of pelvic organ prolapse diagnosis, as all included patients underwent surgery for POP.

**Table 3:** Stratified analysis of effect size—length of hospital stay above the median. *Random Effects Model (DerSimonian–Laird)*.

Variable	Comparison	OR	95% CI	I^2^%	Q	p_het	k	Sign.
Surgical history								
Concomitant total hysterectomy	Yes vs. No	2.05	[1.00–4.20]	43.4%	3.53	0.1710	3	*
Previous hysterectomy	Yes vs. No	1.00	[0.46–2.17]	0.0%	0.82	0.6629	3	
Prior gynecologic surgery	Yes vs. No	0.96	[0.51–1.80]	0.0%	0.03	0.9861	3	
Postoperative complications	Yes vs. No	1.31	[0.59–2.89]	0.0%	1.11	0.5730	3	
Obstetric history								
Neonates > 4 kg	Yes vs. No	2.52	[1.37–4.62]	0.0%	0.56	0.7553	3	*
Multiple pregnancies	Yes vs. No	1.05	[0.06–17.4]	42.9%	1.75	0.1859	2	
Difficult deliveries								
	Forceps vs. No	0.93	[0.36–2.43]	0.0%	1.77	0.4122	3	
	Yes vs. No	1.91	[1.21–3.00]	0.0%	0.54	0.7633	3	*
Menopause	Yes vs. No	1.43	[0.66–3.10]	31.6%	2.93	0.2316	3	
COPD/chronic cough	Yes vs. No	0.64	[0.36–1.13]	0.0%	0.42	0.8120	3	
Heavy lifting	Yes vs. No	0.87	[0.49–1.55]	34.8%	3.07	0.2159	3	
Diabetes mellitus	Yes vs. No	1.28	[0.61–2.66]	0.0%	1.61	0.4463	3	
Stress urinary incontinence (SUI)								
Mixed SUI vs. No SUI		0.58	[0.13–2.65]	0.0%	1.07	0.5856	3	
Pure SUI vs. No SUI		2.09	[0.37–11.8]	0.0%	0.64	0.7269	3	
Associated compartment prolapse severity								
Grade I associated prolapse vs. absence of associated prolapse in that compartment		1.27	[0.58–2.76]	0.0%	1.28	0.5276	3	
Grade II prolapse vs. No prolapse		1.90	[1.04–3.47]	0.0%	0.26	0.8802	3	*
Grade III prolapse vs. No prolapse		3.45	[0.94–12.6]	68.3% *	6.31	0.0426	3	
Grade IV prolapse vs. No prolapse		3.15	[0.90–11.0]	23.0%	2.60	0.2730	3	
Obesity								
Obesity grade I vs. No obesity		1.39	[0.79–2.45]	0.0%	0.88	0.6441	3	
Obesity grade II vs. No obesity		1.67	[0.91–3.05]	0.0%	1.20	0.5484	3	
Obesity grade III vs. No obesity		1.85	[0.75–4.55]	0.0%	0.72	0.6969	3	
Age at admission	Above median (63.0) vs. Below median	1.66	[0.98–2.79]	24.7%	2.66	0.2651	3	
Intraoperative bleeding	Above median (350.0) vs. Below median	1.65	[1.03–2.63]	0.0%	1.85	0.3970	3	*
Number of deliveries	Above median (3.0) vs. Below median	1.73	[1.04–2.86]	0.0%	0.36	0.8371	3	*

OR = pooled Odds Ratio|95% CI = 95% confidence interval|I^2^ = heterogeneity. I^2^ > 50% = moderate heterogeneity|I^2^ > 75% = high heterogeneity. p_het = *p*-value of Cochran’s Q test|* = 95% CI does not include 1 (statistically significant). Variables with I^2^ > 50% and significant Cochran’s Q test were considered heterogeneous; their pooled estimates should be interpreted as exploratory and not as uniform effects across all surgical groups. Colors: yellow = statistically significant association (OR with 95% CI excluding the null value). The term “no prolapse” refers to the absence of associated prolapse in the evaluated compartment, not to the absence of pelvic organ prolapse diagnosis, as all included patients underwent surgery for POP.

**Table 4:** Stratified analysis of effect size—postoperative hospital stay above the median. *Random Effects Model (DerSimonian–Laird)*.

Variable	Comparison	OR	95% CI	I^2^%	Q	p_het	k	Sign.
Surgical history								
Concomitant total hysterectomy	Yes vs. No	1.67	[0.93–2.98]	21.8%	2.56	0.2783	3	
Previous hysterectomy	Yes vs. No	1.24	[0.56–2.74]	0.0%	0.88	0.6425	3	
Prior gynecologic surgery	Yes vs. No	0.67	[0.32–1.39]	4.2%	2.09	0.3520	3	
Postoperative complications	Yes vs. No	2.05	[0.92–4.56]	0.0%	0.77	0.6807	3	
Obstetric history								
Neonates > 4 kg	Yes vs. No	1.89	[1.05–3.41]	0.0%	0.78	0.6786	3	*
Multiple pregnancies	Yes vs. No	1.07	[0.17–6.68]	0.0%	0.65	0.4208	2	
Difficult deliveries								
Forceps vs. No		0.65	[0.21–1.99]	13.2%	2.30	0.3160	3	
Yes vs. No		1.30	[0.54–3.10]	69.0% *	6.45	0.0398	3	
Comorbidities and risk factors								
Menopause	Yes vs. No	1.53	[0.82–2.86]	0.0%	0.76	0.6829	3	
COPD/chronic cough	Yes vs. No	0.94	[0.48–1.86]	26.0%	2.70	0.2588	3	
Heavy lifting	Yes vs. No	1.03	[0.59–1.77]	24.6%	2.65	0.2655	3	
Diabetes mellitus	Yes vs. No	1.02	[0.51–2.07]	0.0%	0.16	0.9244	3	
Stress urinary incontinence (SUI)								
Mixed SUI vs. No SUI		0.84	[0.16–4.27]	0.0%	0.16	0.9222	3	
Pure SUI vs. No SUI		1.54	[0.26–9.07]	0.0%	0.74	0.6914	3	
Associated compartment prolapse severity								
Grade I associated prolapse vs. absence of associated prolapse in that compartment		1.45	[0.65–3.24]	0.0%	0.74	0.6923	3	
Grade II prolapse vs. No prolapse		1.44	[0.78–2.65]	0.0%	1.80	0.4074	3	
Grade III prolapse vs. No prolapse		3.31	[0.51–21.6]	84.8% **	13.19	0.0014	3	
Grade IV prolapse vs. No prolapse		2.98	[1.04–8.51]	0.0%	1.98	0.3709	3	*
Obesity (BMI)								
Obesity grade I vs. No obesity		1.29	[0.71–2.33]	0.0%	0.57	0.7517	3	
Obesity grade II vs. No obesity		1.70	[0.91–3.17]	0.0%	1.12	0.5703	3	
Obesity grade III vs. No obesity		1.21	[0.49–3.00]	0.0%	0.79	0.6748	3	
Continuous variables								
Age at admission	Above median (63.0) vs. Below median	1.48	[0.65–3.34]	67.0% *	6.06	0.0483	3	
Intraoperative bleeding	Above median (350.0) vs. Below median	1.60	[0.83–3.07]	46.5%	3.74	0.1544	3	
Number of deliveries	Above median (3.0) vs. Below median	1.60	[0.86–2.96]	32.3%	2.95	0.2284	3	

OR = pooled Odds Ratio; 95% CI = 95% confidence interval; I^2^ = heterogeneity. I^2^ > 50% was considered moderate heterogeneity, while I^2^ > 75% was considered high heterogeneity. p_het = p-value of Cochran’s Q test. In the Sign column, * indicates that the 95% CI does not include 1, therefore suggesting a statistically significant pooled association. Asterisks placed next to I^2^ values refer to heterogeneity rather than to the pooled effect: * indicates statistically significant heterogeneity at p_het < 0.05, while ** indicates stronger statistical evidence of heterogeneity at p_het < 0.01. Variables with I^2^ > 50% and a significant Cochran’s Q test were considered heterogeneous; therefore, their pooled estimates should be interpreted as exploratory and not as uniform effects across all surgical groups. Colors: yellow = statistically significant association, defined as an OR with 95% CI excluding the null value. The term “no prolapse” refers to the absence of associated prolapse in the evaluated compartment, not to the absence of pelvic organ prolapse diagnosis, as all included patients underwent surgery for POP.

**Table 5:** Stratified analysis of effect size—bladder catheterization days above the median. *Random Effects Model (DerSimonian–Laird)*.

Variable	Comparison	OR	95% CI	I^2^%	Q	p_het	k	Sign.
Surgical history								
Concomitant total hysterectomy	Yes vs. No	0.90	[0.46–1.74]	0.0%	1.34	0.5121	3	
Previous hysterectomy	Yes vs. No	1.78	[0.67–4.71]	0.0%	1.77	0.4131	3	
Prior gynecologic surgery	Yes vs. No	1.19	[0.50–2.85]	0.0%	0.48	0.7847	3	
Postoperative complications	Yes vs. No	6.33	[2.85–14.0]	0.0%	0.13	0.9364	3	*
Obstetric history								
Neonates > 4 kg	Yes vs. No	2.18	[0.98–4.89]	27.3%	2.75	0.2525	3	
Multiple pregnancies	Yes vs. No	0.75	[0.09–6.34]	0.0%	0.40	0.5247	2	
Difficult deliveries								
Forceps vs. No		1.76	[0.55–5.59]	0.0%	1.80	0.4058	3	
Yes vs. No		1.80	[0.50–6.48]	72.3% *	7.21	0.0272	3	
Comorbidities and risk factors								
Menopause	Yes vs. No	1.98	[0.75–5.23]	0.0%	0.16	0.9225	3	
COPD/chronic cough	Yes vs. No	0.81	[0.36–1.83]	0.0%	0.94	0.6251	3	
Heavy lifting	Yes vs. No	0.67	[0.26–1.76]	57.7% *	4.72	0.0942	3	
Diabetes mellitus	Yes vs. No	0.81	[0.30–2.16]	0.0%	0.33	0.8464	3	
Stress urinary incontinence (SUI)								
Mixed SUI vs. No SUI		0.28	[0.05–1.44]	0.0%	0.65	0.7211	3	
Pure SUI vs. No SUI		0.28	[0.04–1.86]	0.0%	0.64	0.7260	3	
Associated compartment prolapse severity								
Grade I associated prolapse vs. absence of associated prolapse in that compartment		4.66	[1.84–11.8]	0.0%	0.06	0.9696	3	*
Grade II prolapse vs. No prolapse		1.14	[0.31–4.20]	38.5%	3.25	0.1969	3	
Grade III prolapse vs. No prolapse		2.46	[0.34–17.6]	71.2% *	6.95	0.0310	3	
Grade IV prolapse vs. No prolapse		2.54	[0.45–14.4]	40.5%	3.36	0.1862	3	
Obesity								
Obesity grade I vs. No obesity		0.99	[0.44–2.27]	0.0%	1.50	0.4731	3	
Obesity grade II vs. No obesity		1.62	[0.73–3.63]	0.0%	0.52	0.7715	3	
Obesity grade III vs. No obesity		1.22	[0.34–4.41]	0.0%	0.57	0.7538	3	
Continuous variables								
Age at admission	Above median (63.0) vs. Below median	2.11	[0.61–7.37]	74.7% *	7.90	0.0193	3	
Intraoperative bleeding	Above median (350.0) vs. Below median	1.03	[0.49–2.18]	33.1%	2.99	0.2242	3	
Number of deliveries	Above median (3.0) vs. Below median	2.52	[1.35–4.69]	5.2%	2.11	0.3482	3	*

OR = pooled Odds Ratio|95% CI = 95% confidence interval|I^2^ = heterogeneity. I^2^ > 50% = moderate heterogeneity|I^2^ > 75% = high heterogeneity. p_het = *p*-value of Cochran’s Q test|* = 95% CI does not include 1 (statistically significant). Variables with I^2^ > 50% and significant Cochran’s Q test were considered heterogeneous; their pooled estimates should be interpreted as exploratory and not as uniform effects across all surgical groups. Colors: yellow = statistically significant association (OR with 95% CI excluding the null value). The term “no prolapse” refers to the absence of associated prolapse in the evaluated compartment, not to the absence of pelvic organ prolapse diagnosis, as all included patients underwent surgery for POP.

**Table 6:** Multiple linear regression—effect of surgical procedures on continuous perioperative outcomes.

Model 1: Length of hospital stay (D) R^2^ = 0.0619 R^2^adj = 0.0569 F = 12.306 *p* ≤ 0.0001 *** N = 376							
**Predictor**	**Coef**	**SE**	**t**	***p***	**95% CI**	**Beta std**	**Sign.**
SFM + TOT/TVT (ref)	0	—	—	—	[0; 0]	0	ref
A&P repair + TOT/TVT	0.6553	0.3279	1.9980	0.0464	[0.0105; 1.3001]	0.1288	*
A&P repair	1.7108	0.3573	4.7880	<0.0001	[1.0083; 2.4134]	0.3086	***
Model 2: Postoperative days (E) R^2^ = 0.0801 R^2^adj = 0.0751 F = 16.229 *p* ≤ 0.0001 *** N = 376							
**Predictor**	**Coef**	**SE**	**t**	***p***	**CI 95%**	**Beta std**	**Sign.**
SFM + TOT/TVT (ref)	0	—	—	—	[0; 0]	0	ref
A&P repair + TOT/TVT	0.6903	0.2465	2.8000	0.0054	[0.2056; 1.1750]	0.1787	**
A&P repair	1.5092	0.2686	5.6200	<0.0001	[0.9811; 2.0373]	0.3587	***
Model 3: Bladder catheterization days (F) R^2^ = 0.0592 R^2^adj = 0.0542 F = 11.745 *p* ≤ 0.0001 *** N = 376							
**Predictor**	**Coef**	**SE**	**t**	***p***	**CI 95%**	**Beta std**	**Sign.**
SFM + TOT/TVT (ref)	0	—	—	—	[0; 0]	0	ref
A&P repair + TOT/TVT	0.5358	0.1866	2.8710	0.0043	[0.1689; 0.9028]	0.1853	**
A&P repair	0.9844	0.2033	4.8420	<0.0001	[0.5846; 1.3842]	0.3125	***
Model 4: Intraoperative bleeding (mL) (CA) R^2^ = 0.0353 R^2^adj = 0.0301 F = 6.820 *p* = 0.0012 ** N = 376							
**Predictor**	**Coef**	**SE**	**t**	***p***	**CI 95%**	**Beta std**	**Sign.**
SFM + TOT/TVT (ref)	0	—	—	—	[0; 0]	0	ref
A&P repair + TOT/TVT	−14.4545	10.8823	−1.3280	0.1849	[−35.8528; 6.9438]	−0.0868	ns
A&P repair	21.9000	11.8568	1.8470	0.0655	[−1.4145; 45.2145]	0.1207	ns

Multiple linear regression—four models|Independent variable: surgical procedures|Reference: SFM + TOT/TVT. Coef = unstandardized coefficient|SE = standard error|95% CI = confidence interval|Std Beta = standardized coefficient. *** *p* < 0.001|** *p* < 0.01|* *p* < 0.05|ns = not significant|ref = reference category. Yellow = statistically significant predictor|Grey = reference category|Positive coefficient = higher value compared with the reference group.

## Data Availability

The data presented in this study are available on request from the corresponding author. The data are not publicly available due to privacy and ethical restrictions.

## Abbreviations

SFM	Sacrospinous Fixation Mesh
TOT	Transobturator Tape
TVT	Tension-Free Vaginal Tape
POP	Pelvic Organ Prolapse
SUI	Stress Urinary Incontinence
UTI	Urinary Tract Infection
BMI	Body Mass Index
OR	Odds Ratio
CI	Confidence Interval
SD	Standard Deviation
ANOVA	Analysis of Variance
I^2^	I-squared Statistic
Q	Cochran’s Q Statistic
